# Intra-species Genomic and Physiological Variability Impact Stress Resistance in Strains of Probiotic Potential

**DOI:** 10.3389/fmicb.2018.00242

**Published:** 2018-02-20

**Authors:** Jason W. Arnold, Joshua B. Simpson, Jeffrey Roach, Jakub Kwintkiewicz, M. Andrea Azcarate-Peril

**Affiliations:** ^1^Division of Gastroenterology and Hepatology, Department of Medicine, Microbiome Core Facility, Center for Gastrointestinal Biology and Disease, School of Medicine, University of North Carolina, Chapel Hill, NC, United States; ^2^Department of Chemistry, College of Arts and Sciences, University of North Carolina, Chapel Hill, NC, United States; ^3^Research Computing, University of North Carolina, Chapel Hill, NC, United States

**Keywords:** probiotics, intra-Species variability, bacterial stress, *Lactobacillus rhamnosus*, bile salt hydrolase (BSH)

## Abstract

Large-scale microbiome studies have established that most of the diversity contained in the gastrointestinal tract is represented at the strain level; however, exhaustive genomic and physiological characterization of human isolates is still lacking. With increased use of probiotics as interventions for gastrointestinal disorders, genomic and functional characterization of novel microorganisms becomes essential. In this study, we explored the impact of strain-level genomic variability on bacterial physiology of two novel human *Lactobacillus rhamnosus* strains (AMC143 and AMC010) of probiotic potential in relation to stress resistance. The strains showed differences with known probiotic strains (*L. rhamnosus* GG, Lc705, and HN001) at the genomic level, including nucleotide polymorphisms, mutations in non-coding regulatory regions, and rearrangements of genomic architecture. Transcriptomics analysis revealed that gene expression profiles differed between strains when exposed to simulated gastrointestinal stresses, suggesting the presence of unique regulatory systems in each strain. *In vitro* physiological assays to test resistance to conditions mimicking the gut environment (acid, alkali, and bile stress) showed that growth of *L. rhamnosus* AMC143 was inhibited upon exposure to alkaline pH, while AMC010 and control strain LGG were unaffected. AMC143 also showed a significant survival advantage compared to the other strains upon bile exposure. Reverse transcription qPCR targeting the bile salt hydrolase gene (*bsh*) revealed that AMC143 expressed *bsh* poorly (a consequence of a deletion in the *bsh* promoter and truncation of *bsh* gene in AMC143), while AMC010 had significantly higher expression levels than AMC143 or LGG. Insertional inactivation of the *bsh* gene in AMC010 suggested that *bsh* could be detrimental to bacterial survival during bile stress. Together, these findings show that coupling of classical microbiology with functional genomics methods for the characterization of bacterial strains is critical for the development of novel probiotics, as variability between strains can dramatically alter bacterial physiology and functionality.

## Introduction

The gastrointestinal tract is host to one of the densest and most diverse microbial communities on the planet (Gill et al., 2006; Li et al., 2012). The composition and function of the gut microbiota is critical to maintenance of host gastrointestinal health (Jones, 2016). In fact, diseases including diabetes (Tilg and Moschen, 2014), obesity (Carding et al., 2015), and colorectal cancer (Sobhani et al., 2011; Borges-Canha et al., 2015) have been shown to have gut dysbioses associated with progression. Conversely, modulation of the gut microbiota can alleviate or even eliminate disorders like *Clostridium difficile* infections (Petrof et al., 2013; Allegretti et al., 2014), inflammatory bowel disease (D'Haens et al., 2014), and lactose intolerance (Azcarate-Peril et al., 2017).

Intra-species genetic polymorphisms constitute the majority of the diversity within the microbiota of the human gut (Greenblum et al., 2015; Zhang and Zhao, 2016). Coupling classical microbiology approaches with next generation sequencing provides an opportunity to study physiological characteristics of individual microbial strains, and to identify unique genomic elements associated with those phenotypes. Moreover, advances in cultivation technologies have improved isolation of novel microorganisms from human subjects, provided the ability to study physiology of difficult to grow microbes (Faith et al., 2010), and ultimately develop advanced microbiota-derived treatments for gastrointestinal diseases (Forster and Lawley, 2015).

Use of probiotics, live microbes that convey a benefit to their host when administered in adequate amounts, as interventions for gastrointestinal disorders has gained increasing support (Grover et al., 2012; Vandenplas et al., 2015); however, intra-species variations can impact their functionality as effective probiotics (Chapman et al., 2012). Isolation and characterization of novel intestinal organisms are important steps toward the development of new and improved probiotics. Moreover, high throughput sequencing technology allow for cost-effective whole genome sequencing of bacterial isolates, providing a wealth of genomic data. Nevertheless, identification and characterization of novel probiotics also require careful examination of microbial physiology (Papadimitriou et al., 2015).

One essential physiological characteristic of probiotics is their ability to survive the environmental conditions of the gastrointestinal tract. Gut microorganisms have evolved highly conserved mechanisms for tolerance to gastrointestinal stresses, which include variations in pH and the antimicrobial effects of bile (Azcarate-Peril et al., 2004; Bruno-Bárcena et al., 2004). These mechanisms are controlled by well-regulated genetic systems (Azcarate-Peril et al., 2004), often with rapid response times to acute stresses, allowing for immediate resistance to environmental changes (Anderson et al., 2010; Bove et al., 2013). Bacterial responses to environmental stress occur in a highly regulated manner. First, stress-specific genes aim to aleviate the immediate stress (maintenance of cytoplasmic pH homeostasis, transport/degradation of toxic compounds, and others), followed by universal stress response systems aimed to repair DNA, protein, membrane, and cell wall damage.

If exposed to an acidic environment, H^+^ pump of ATPases (F_0_F_1_-ATPases) maintain pH homeostasis within the cytoplasm by pumping excess H^+^ ions back into the environment (Wang et al., 2013; Cusumano and Caparon, 2015). Other mechanisms involved in resistance to acid stress in *Lactobacillus* are arginine deiminases (ArcABC) (Fulde et al., 2014) that synthesize ammonia from arginine and free H^+^ within the cytoplasm (Casiano-Colón and Marquis, 1988) and amino acid decarboxylases coupled with amino acid/biogenic amines antiporter systems (Azcarate-Peril et al., 2004; Papadimitriou et al., 2016). Upon surviving the acidic conditions in the stomach and passing into the duodenum, the pH of the environment shifts from highly acidic to alkaline. Alkaline Shock Proteins (Asp) are encoded by lactic acid bacteria including *Lactobacillus rhamnosus* (Arnold et al., 2017), and have been implicated in resistance to sudden pH shifts (Kuroda et al., 1995; Seetharaaman, 2008; Anderson et al., 2010).

Entry into the duodenum not only involves a change in pH, but also exposes microorganisms to bile salts, which act as detergents, causing cell damage and cytotoxicity (Andreichin, 1980; Begley et al., 2005). Lactic acid bacteria including *Lactobacillus* and *Bifidobacterium* species encode bile salt hydrolases (*bsh*), which have been shown in some cases to provide resistance to bile toxicity (Grill et al., 2000; Patel et al., 2010; Lin et al., 2014). *Lactobacillus acidophilus* and *Bifidobacterium* encode multiple *bsh* genes, which exhibit substrate specificity for different conjugated bile salt targets (McAuliffe et al., 2005; Jarocki and Targonski, 2013). As different bile salts have varying toxicity, individual *bsh* genes may or may not provide survival advantages against bile (Fang et al., 2009). In addition to providing survival advantages in the gastrointestinal environment, bile salt hydrolases encoded by lactic acid bacteria (Jarocki and Targonski, 2013; Jarocki et al., 2014; Pithva et al., 2014) have been implicated in modulation of host cholesterol and lipid metabolism (Patel et al., 2010; Joyce et al., 2014), and as a mechanism for microbe-host signaling (Ridlon et al., 2006).

Universal bacterial stress-response systems encode mechanisms to cope with the deleterious consequences of exposure to environmental stresses, including DNA damage, protein misfolding, and loss of cell wall/membrane integrity. Bacterial DNA damage is repaired by MutS and RecA-like recombinases (Lee and Pi, 2010; Rossi et al., 2016; Calderini et al., 2017; Overbeck et al., 2017). Protein misfolding is reduced and repaired by molecular chaperones including heat-shock proteins (Ruiz et al., 2013; Calderini et al., 2017). Cell wall integrity of gram positive bacteria is maintained through lipoteichoic acid (LTA), exopolysaccharide, and fatty acid metabolism/synthesis genes (Koskenniemi et al., 2011). Membrane proteins including metalloproteases provide additional stress resistance in lactic acid bacteria (Bove et al., 2012). *Lactobacillus* also encodes two component regulatory systems (2CRS) that promote rapid responses to environmental stresses. These systems usually consist of a histidine kinase and a response regulator gene that work to sense and react to changes in the environment (Yu et al., 2014; Monedero et al., 2017), facilitating stress resistance (Morel-Deville et al., 1998; Alcántara et al., 2011). Together, universal stress-response genes work with specific stress resistance systems to ensure bacterial survival to gastrointestinal conditions.

Douillard et al. (2013) in their analysis of 100 *L. rhamnosus* genomes, showed that strains had unique genomic elements coupled with physiological traits that were influenced by the strain origin. In particular, bile resistance was higher in *L. rhamnosus* isolates derived from the gastrointestinal tract as opposed to dairy, oral, or vaginal isolates. In this study we analyzed genomic, transcriptomic, and physiological characteristics of two novel human *L. rhamnosus* strains of probiotic potential for which our group recently generated genomic sequence information (Thompson et al., 2015; Arnold et al., 2017), and compared them with an established probiotic strain of human origin (LGG). *Lactobacillus rhamnosus* GG (LGG) is a well-characterized probiotic lactic acid bacteria isolated from a healthy human host in 1983. The strain provides a number of benefits to its host including immunomodulation (Lebeer et al., 2012; Segers and Lebeer, 2014), pathogen exclusion (De Keersmaecker et al., 2006; Makras et al., 2006), and modulation of host gene expression (Kankainen et al., 2009; Yan et al., 2013; Segers and Lebeer, 2014). LGG sets a precedent for *L. rhamnosus* as a functional probiotic as well as for the isolation and identification of novel probiotics from healthy human hosts. Additionally, the strains were compared with *L. rhamnosus* of dairy origin (Lc705, HN001; Ceapa et al., 2015) to demonstrate the impact of intra-species genetic polymorphisms on tolerance to gastrointestinal stress.

## Materials and methods

### Strains and culture media

Bacterial strains used in this study are presented in Table 1. Strains were propagated in MRS broth (Pronadisa, Madrid) at 37°C without agitation, or on MRS agar plates containing 1.5% agar. For growth assays, MRS supplemented with 0.1–1.0% Oxgall was used to test resistance to bile. MRS was titrated with HCl–pH 4 to test growth in acidic conditions, and titrated with NaOH to pH 8 to test growth under alkaline stress.

**Table 1:** Bacterial strains used in this study.

**Strain**	**Origin**	**References**
*L. rhamnosus* GG	Healthy human isolate	Kankainen et al., 2009
*L. rhamnosus* Lc705	Fermented dairy product	Kankainen et al., 2009
*L. rhamnosus* HN001	Fermented dairy product	Tannock et al., 2014
*L. rhamnosus* AMC010	Human infant stool	Arnold et al., 2017
*L. rhamnosus* AMC010::*bsh*		This study
*L. rhamnosus* AMC143	Human infant stool	Arnold et al., 2017

### Bacterial growth assays

*Lactobacillus rhamnosus* strains were grown statically for 16 h in MRS broth at 37°C. Freshly harvested cells were washed with MRS without glucose and diluted 1:100 in MRS (pH 6.6) containing either bile (0, 0.1, 0.3, 0.5, and 1.0% w/v oxgall), or adjusted to pH 4 (HCl) or pH 8 (NaOH). 200 μl of bacterial suspensions were placed into each well of a 96 well plate, sealed and placed into Tecan Infinite 200 Pro spectrophotometer (Tecan, Switzerland), where they were grown for 24 h at 37°C. Measurements of optical density at 600 nm (OD_600nm_) were taken every 15 min during this 24-h period. Maximum specific growth rates (μ_max_) were then calculated for each treatment type and plotted in Origin2016 (OriginLab, Northampton, MA).

### Bacterial survival assays

Bacterial cells grown to mid-log growth phase (OD_600nm_ = ~0.6) were centrifuged, washed twice with sterile PBS and diluted 1:10,000 in simulated gastric juice (0.5% pepsin, 0.5% NaCl, pH3), simulated intestinal juice (0.3% pancreatin, 0.5% NaCl, pH8), or bile (0.5% Oxgall in MRS + 0% Glucose). Cell suspensions were incubated for 2 h at 37°C and then plated onto MRS agar plates by WASP Spiral Plater (Don Whitley Scientific, West Yorkshire, UK) at 1 and 2 h post inoculation. Plates were incubated at 37°C for 48 h. Survival was measured using ProtoCOL colony counter (Synbiosis, Frederick, MD) to count colonies present on MRS plates of treated and untreated cells. Three biological replicates with three technical replicates were included in survival experiments. Pairwise comparisons using a two-tailed Student's *t*-test were performed to determine statistically significant differences between treatments.

### RNA isolation

AMC010, AMC143, and LGG were grown to early log phase (OD_600nm_ = ~0.4) in MRS prior to harvesting. Cells were then washed twice with sterile PBS and re-suspended in simulated gastric juice, simulated intestinal juice, or bile (0.5% w/v Oxgall in sterile PBS) and incubated for 10 min at 37°C. Three biological replicates were included in expression experiments. After incubation, cells were centrifuged, flash frozen in RNAlater (Thermo Fisher Scientific, Waltham, MA), and stored in −80°C until RNA isolation was performed. RNA isolation was performed using the Qiagen RNeasy PowerMicrobiome kit (Qiagen, Valencia, CA) as directed. RNA was eluted in 50 μl RNase free water and quantified using the 2200TapeStation (Agilent Technologies, Santa Clara, CA).

### Real time quantitative PCR

Five ng of total RNA isolated from bacterial cultures exposed to bile (0.5% w/v Oxgall in sterile PBS for 30 min at 37°C) were reverse transcribed using qScript cDNA SuperMix (Quanta BioSciences, Gaithersburg, MD). To generate a standard curve, genomic DNA from AMC010 was amplified using primer set BSHqPCR-F (TTGGCGCTGACGACTTGC), BSHqPCR-R (AATCTTGACGCCTTGACC) (this study) and purified via gel extraction in 1.5% agarose gel. The purified PCR product was serially diluted and used in RT–qPCR experiments. The qPCR master mix included 10 μl of Power SYBR® Green 2x PCR Master Mix (Applied Biosystems, Foster City, CA), 2 μl of each primer (BSHqPCR-F, BSHqPCR-R) (1 μM stock), 1 μl PCR grade water, and 5 μl of template cDNA (1 ng/μl). The reaction was run for 40 cycles of melting (95°C) 15 s, annealing/extension (65°C) 45 s followed by SYBR detection was carried out on the 7,500 Fast Real Time PCR System (Applied Biosystems, Foster City, CA) after 10 min denaturation step at 95°C. Samples and standards were run in triplicate.

### mRNA sequencing

Ribosomal RNA was depleted from total RNA using the Ribo-Zero Gold Bacterial rRNA Removal Reagent (Epidemiology Kit) (Illumina, San Diego, CA) according to manufacturer's instructions. Briefly, the rRNA-specific magnetic beads were removed from storage buffer and mixed with 500 ng of total sample RNA. Subsequently, rRNA removal solution was added and samples were incubated for 10 min at 65°C. Finally, samples were placed on magnetic stand for 15 min at 22°C and mRNA was removed and immediately processed with TruSeq Stranded mRNA HT kit (Illumina, San Diego, CA) according to manufacturer's instructions. Briefly, RNA was mixed with Fragment-Prime mix and incubated at 94°C for 8 min. Samples were immediately subject to first strand and second strand cDNA synthesis reactions, respectively, followed by 3′ end repair, adenylation and adapter ligation. After adapter ligation, the libraries were enriched by PCR using the following thermal cycling conditions: 98°C for 30 s followed by 15 cycles of 98°C for 10 s, 60°C for 30 s and 72°C for 30 s. Final extension step of 70°C for 5 min was carried out following the last cycle. After enrichment, libraries were purified with Beckman Coulter magnetic beads (Brea, CA), washed with 80% ethanol and eluted in Tris pH 8.5. Following enrichment, cDNA was barcoded for multiplexing via PCR, using dual-index barcodes [index 1(i7) and index 2(i5)] (Illumina, San Diego, CA) in a combination unique to each sample. Final ds cDNA was purified with Beckman Coulter magnetic beads (Brea, CA). Library concentrations and quality were measured via TapeStation2200 (Agilent Technologies, Santa Clara, CA). Barcoded libraries were pooled at equimolar concentrations and sequenced on the Illumina HiSeq platform (Illumina, San Diego, CA).

### mRNA sequencing data analysis

Sequencing output from the Illumina HiSeq platform was converted to FASTQ format and demultiplexed using Illumina BclFastq 2.18.0.12 (Illumina, San Diego, CA). Quality control was performed via FastQC on both raw and processed sequencing reads (Babrahm Institute, Cambridge, UK). Demultiplexed FASTQ sequence files were uploaded to Geneious software (Biomatters, New Zealand). Sequencing reads from each treatment were mapped against LGG genome in Geneious software using “Geneious for RNA Seq” mapper, a minimum mapping quality of 30 (99.9% confidence), allowing gaps with a maximum of 10% per read, 20% maximum mismatches per read, with word length of 20 bases. Expression levels were calculated in Geneious and compared between treatment types. Genes identified as significantly differentially regulated between treatments (*p* ≤ 0.05) were further filtered to include only genes with 2-fold expression differences. Genes identified as significantly differentially regulated at ≥2-fold in at least one treatment type for each isolate were plotted as heat maps in OriginLab software (Origin Lab, Northampton, MA), and compared between strains to show differences.

### Preparation of electrocompetent cells of *L. rhamnosus*

Electrocompetent *L. rhamnosus* cells were generated using a combination of previously described methods (Kim et al., 2005; Welker et al., 2015), with minor adjustments. Briefly, an overnight culture of *L. rhamnosus* AMC010 was diluted to 10^6^ cells/ml in pre-warmed MRS broth supplemented with 2% glycine and was incubated overnight at 37°C without agitation. After incubation, 5 ml of culture was inoculated in 100 ml of freshly prepared, pre-warmed MRS medium supplemented with 2% glycine. The culture was incubated at 37°C without agitation until it reached and OD_600nm_ of 0.2. Ampicillin was then added to the culture at a final concentration of 10 μg/ml, and incubation at 37°C continued until the culture reached an OD_600nm_ of 0.4. Cells were then harvested by centrifugation at room temperature (10 min at 6,000 g), washed twice at room temperature with electroporation buffer (0.5 M sucrose, 7 mM potassium phosphate pH7.4, 1 mM MgCl_2_), resuspended in 1 ml of the same buffer, and placed on ice. The electrocompetent cells were used immediately for electroporation.

### Generation of the *bsh* insertional mutant strain

Insertional inactivation of the *bsh* gene in *L. rhamnosus* AMC010 was done as described (Welker et al., 2015). Briefly, primers BshFHindIII (TATTAAGCTTTTCAGACAGAGGCGGCTTTGC) and BshREcoRI (AATAGAATTCAATCTTGACGCCTTGACCAC) were designed to amplify a 652 bp region of the *bsh* gene in AMC010. The primers included EcoR1 and HindIII restriction sites for cloning into the pFAJ-5301 vector (Lebeer et al., 2012). The resulting vector (pFAJ-BSHi) was used to transform AMC010 by electroporation using an Bio-Rad Gene Pulser (peak voltage, 1.7 kV; capacitance, 25 μF; resistance, 200 Ω with time constant of 2–4 ms). Electroporated AMC010 cells were allowed to recover for 24 h in MRS at 37°C without agitation, and subsequently plated on MRS containing erythromycin at a final concentration of 2 μg/ml. Individual erythromycin resistant colonies were selected after 48 h of growth at 37°C and sub-cultured in MRS containing 2 μg/ml erythromycin overnight without agitation at 37°C. Disruption of the *bsh* genes was verified by PCR amplification using primers Bsh1753F (ATTGCCTGACCTAGATGCAGG) and Bsh1753R (AACACCGGCGACAGGTCCATC). Genomic integration of the erythromycin resistance cassette was also verified by PCR amplification with the primers Ery625F (CTACTTAATCTGATAAGTGAGC) and Ery625R (TCAGCACAGTTCATTATCAACC). AMC010::*bsh* mutants were cultivated in MRS broth containing 2 μg/ml erythromycin and stored at −80°C in 15% glycerol.

## Results

### Comparative analysis of the *L. rhamnosus* stress genomic complement

**Figure 1:**
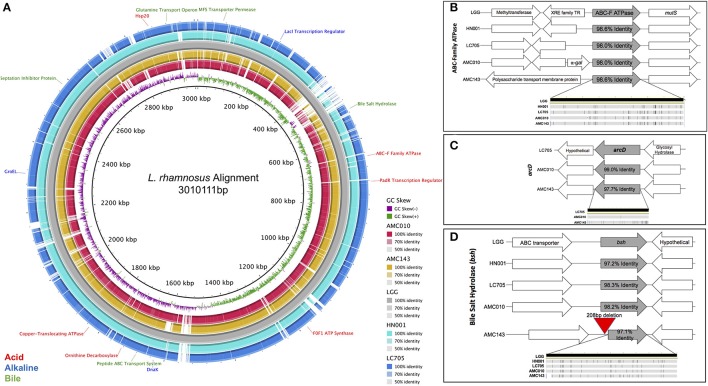
Comparative genomic analysis of *Lactobacillus rhamnosus* isolates. **(A)** Genome alignment of *L. rhamnosus* strains. Strains AMC43, AMC010, Lc705, and HN001 were compared to LGG using BRIG genome alignment software. Relevant stress-response genes are annotated. Comparison of relevant stress-response genes in *L. rhamnosus*, **(B)** ABC-F ATPase, **(C)** *arc*D, **(D)** *bsh* were aligned and compared in Geneious 10.1.3 software. The bottom figure in each graph represents single nucleotide polymorphisms within the compared gene.

The genomes of *L. rhamnosus* AMC 143 and AMC010 isolated from infant stools (Thompson et al., 2015; Arnold et al., 2017) were aligned to the genome of the well-characterized probiotic *L. rhamnosus* GG (Segers and Lebeer, 2014) for comparison (Figure 1A). Our study focused on stress genes and systems encoded by AMC143 and AMC010 in comparison with other *L. rhamnosus* strains of dairy and human origin.

When exposed to environmental pH fluctuations, proton translocating F_0_F_1_-ATPases are the primary genes responsible for maintaining cytoplasmic pH homeostasis (Azcarate-Peril et al., 2004). Each strain in this study encoded ATPase genes with high nucleotide identity to one another (>98%), however the genomic architecture surrounding the ABC-F Family ATPase varied between strains (Figure 1B). In addition to ATPases, cytoplasmic pH homeostasis can be maintained by amino acid decarboxylases and their corresponding antiporters (Azcarate-Peril et al., 2004). Both AMC010 and AMC143 encoded the arginine/ornithine antiporter *arcD* gene; however, the 12 kb region that included this unique *arc*D gene was absent in LGG and HN001 (Figure 1C). This region encompassed *arc*D as well as an NADH oxidase, two diguanylate cyclases, cellulose synthase, glycosyl transferase, glycosyl hydrolase and a hypothetical protein. An ornithine decarboxylase was identified in all strains with over 97% nucleotide identity with no architectural deviations between strains. Another amino acid decarboxylase and antiporter system, the glutamate decarboxylase/glutamate gamma-aminobutyrate antiporter (*gad*C) has been shown to reduce the impact of acid stress in *Lactobacillus* (Azcarate-Peril et al., 2004); however, the *gad*C gene, as well as the corresponding decarboxylase gene were absent in both AMC010 and AMC143. Finally, an uncharacterized amino acid decarboxylase gene was present in LGG (LGG_RS12751) with 97.2% nucleotide identity to its homolog in AMC010, but this gene was absent in AMC143.

Similarly to acid exposure, cytoplasmic pH homeostasis is critical to maintain when cells are exposed to alkaline stress. Sodium-proton antiporters and potassium-proton antiporters maintain cytoplasmic pH in alkaline extracellular environments (Lee et al., 2011; Nyanga-Koumou et al., 2012). The strains in this study encoded 3 sodium-proton antiporters (98.2, 98, and 97.1% nucleotide identity). LGG encoded a unique sodium-proton antiporter absent in AMC010 and AMC143. Each strain also encoded a single potassium transporter, with 97.3% nucleotide identity between strains. Alkaline shock proteins (Asp) have been implicated in providing protection from damage caused by increases in extracellular pH change (Kuroda et al., 1995; Seetharaaman, 2008; Anderson et al., 2010). Each of the strains in this study encoded 4 *asp* genes including *asp23*, each with minimum of 98.3% nucleotide identity between strains.

The anti-microbial/detergent properties of bile salts require cells to respond upon exposure in order to resist cytotoxic effects. Each strain in this study encoded a single copy of a bile salt hydrolase (*bsh*). This gene has been implicated in bile-stress tolerance in other lactobacilli (McAuliffe et al., 2005). The sequence identity of the *bsh* gene exceeded 98.2% between strains, however AMC143 contained a deletion of 208 base pairs upstream of the *bsh* gene, eliminating a putative promoter, ribosome binding site, and inducing a 38 bp truncation of the *bsh* gene itself (Figures 1D, **5B**). Aside from the deletion in AMC143, the *bsh* upstream non-coding region has 93% nucleotide identity between strains. Each strain also encoded cell wall synthesis/repair pathways including DltABCD (Koskenniemi et al., 2011).

In addition to specific stress-response mechanisms, universal stress response systems were present in AMC143 and AMC010. Genes associated with DNA repair were highly conserved, including *radA, recN, recO, recU, rad*C, *recA*, and *mut*S, each of which had over 96.5% nucleotide identity between strains. Additionally, each strain encoded a suite of highly conserved molecular chaperones, which mitigate stress-associated protein misfolding. These genes included *dnaK, groEL, groES, HSP20, HSP33, dnaI*, and *clpB*, each exhibiting 98.2% nucleotide identity or higher between strains. Finally, bacteria membrane integrity is often compromised as a result of environmental stress, allowing metal ions otherwise excluded from the cytoplasm to permeate through the membrane, causing further DNA damage and cell death (Pratviel, 2012; Qiao and Ma, 2013). One mechanism that bacteria have to survive declines in membrane integrity is to actively pump cytotoxic metal ions out of their cytoplasm utilizing metal translocating ATPases (Chien et al., 2013). Each strain encoded three different non-specific metal transporting ATPases each with 97.6% nucleotide identity between strains, as well as specific ATPases for transport of copper (97.0% nucleotide identity between strains) and magnesium (97.5% nucleotide identity between strains).

### Gene expression analysis under stress conditions

**Figure 2:**
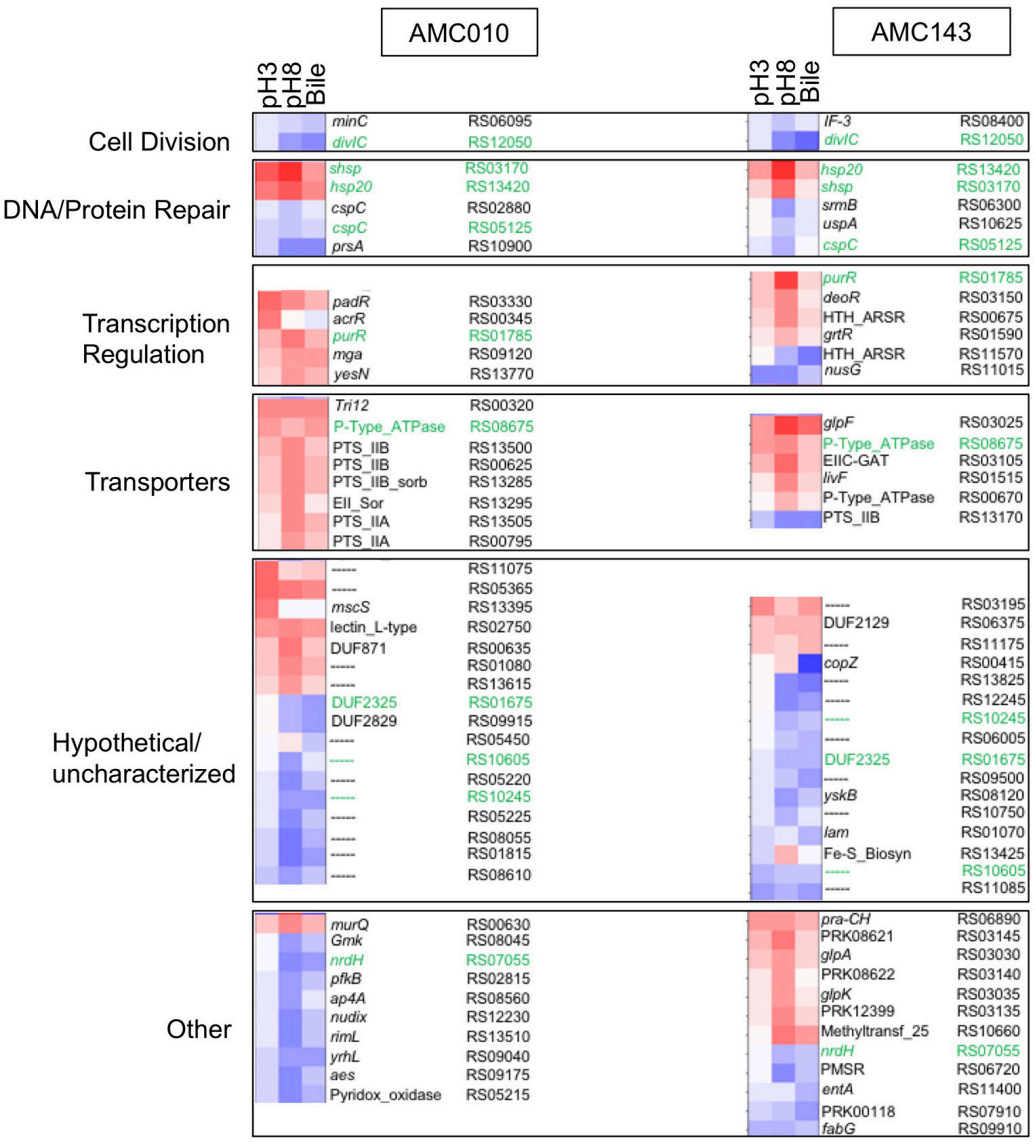
Transcriptomics analysis. mRNA sequencing data from each treatment (pH3, pH8, 0.5%, w/v Bile) was mapped to LGG genome and compared to untreated cells to calculate Log2 differential expression ratio and *p*-value. The 50 genes with the highest differential expression ratio (FDR corrected *p* ≤ 0.05) from each strain were categorized by function (cell division, DNA/protein repair, transcription regulation, and molecular transport) and plotted in OriginLab software. Highly differentially expressed hypothetical genes and genes with functions that did not fit into a defined category were also included. Green text highlights genes differentially regulated in both AMC010 and AMC143.

Differential transcription profiles were generated for AMC010 and AMC143 from mRNA sequencing data obtained from cells exposed to acid (pH 3 adjusted with HCl), alkaline (pH8 adjusted with NaOH), or bile (0.5% w/v oxgall in PBS) stress, and compared to untreated cells. A total 216 genes were identified as differentially regulated (>2-fold change, *p* < 0.05) for AMC010 in response to all treatments, while 79 genes were identified in AMC143. Figure 2 shows a heatmap indicating genes that were up or down regulated in each strain. We mapped our strains to *L. rhamnosus* GG to provide gene homolog references. Our data confirmed differential regulation of universal stress-response genes and specific stress response genes, although most specific genes were differentially regulated below either the statistical or fold change cutoffs (Supplementary Table 1).

Exposure to pH 3 increased expression of a copper-translocating P-type ATPase homologous to LGG_RS08675 by approximately 4-fold in both strains. F_0_F_1_-ATPases were not differentially regulated between strains, however a metal transporting ATPase homologous to LGG_RS00670 was up-regulated approximately 1.5-fold in both AMC010 and AMC143 upon acid exposure. Amino acid decarboxylases were not differentially regulated between strains. All treatments induced expression of a gene encoding a small heat shock protein Hsp20 homologous to LGG_RS13420 in both AMC143 and AMC010. Exposure of AMC010 to acid induced expression of an additional uncharacterized Hsp20-like molecular chaperone (LGG_RS03170, 11.3-fold), the *padR* transcription regulator (LGG_RS03330, 7.5-fold), and two hypothetical proteins (uncharacterized, *Lactobacillus*-specific LGG_RS11075, 9.2-fold, and *fliK*-like LGG_RS13395, 6-fold). Conversely, a gene cluster including 5 genes involved in purine biosynthesis (LGG_RS08715, LGG_RS08720, LGG_RS08725, LGG_RS08730, and LGG_RS08735) was inhibited in AMC010 across all conditions.

Exposure to alkaline conditions induced expression of the alkaline-shock protein Asp23/Gls24 family envelope stress response protein homologous to LGG_RS01130 in AMC010 by 2.3-fold. This gene also showed a non-statistically significant 2-fold induction in AMC143. Other alkaline shock proteins homologous to LGG_RS01125 and LGG_RS08100 were induced by at least 2-fold in both strains, while the putative alkaline shock protein homologous to LGG_RS01130 was repressed by approximately 2-fold under the same condition, though *p*-values for each of these genes fell below the significance cutoffs set for this study. Alkaline stress also induced the *lacI* transcription regulator (homologous to LGG_RS01785) in both AMC010 (6.1-fold) and AMC143 (12.1-fold), the Hsp20-like molecular chaperone (LGG_RS03170, 19.7-fold in AMC143), which was also induced by exposure to acid in AMC010, and uncharacterized hypothetical proteins homologous to LGG_RS00635 (7-fold) and LGG_RS01080 (5.7-fold) in AMC010. Additionally, exposure to pH 8 resulted in decreased expression of the translation initiation factor IF-3 (LGG_RS08400) and a ribosomal-processing cysteine protease (Prp) homologous to LGG_RS08120, reducing expression levels by 2.6-fold and 4.3-fold respectively in AMC143. Alkaline challenge of AMC010 resulted in reduced expression of the septation inhibitor protein similar to *divIC* (Bennett et al., 2007) (LGG_RS12050) by 3.7-fold, and *minC/ftsL*, genes associated with inhibition of cell division (LGG_RS06095 by 2-fold and LGG_RS06140 by −3.5-fold) ABC transport ATP binding protein (LGG_RS09570) by 4.3-fold and a YbjQ_1 domain-containing hypothetical protein homologous to LGG_RS02955 by 42.2-fold. The galactose-6-phosphate isomerase operon (LGG_RS03135, LGG_RS03140, LGG_RS03145, and LGG_RS03150) was specifically up regulated in AMC143 exposed to pH 8, while the Rpml/translation initiation factor gene cluster (LGG_RS08400 and LGG_RS08395) was down regulated in the same strain by approximately 3-fold.

Exposure to bile resulted in the differential expression of 52 genes in AMC143 and 111 genes in AMC010 with considerable overlap with alkaline treatment. Although the *bsh* gene has been shown to be induced by exposure to bile in *Lactobacillus* (Koskenniemi et al., 2011), in our study, *bsh* (similar to LGG_RS02395) showed a non-statistically significant (*p* = 0.2) 1.2-fold induction in AMC010 while AMC143 showed a marginal repression. The genes more impacted by bile exposure in AMC143 were a HU-family transcription regulator homologous to LGG_RS06660 (induced 2-fold), a septation inhibitor protein (LGG_RS12050), which was down regulated 9-fold, the glutamine ABC transport system operon composed of genes homologus to LGG_RS13875, LGG_RS13880, and LGG_RS13885, which were down regulated 2-fold, and hypothetical proteins homologous to LGG_RS01070, LGG_RS09500, and LGG_RS00415, down regulated 2.8-, 4-, and 13.9-fold respectively.

Exposure of AMC010 to Oxgall resulted in a 2.8-fold induction of a 2CRS response regulator (RR), *dcu*R (homologous to LGG_RS13770), similar to the RR involved in regulation of the anaerobic fumarate respiratory system in *E. coli* (Janausch et al., 2004), as well as a 5.3-fold induction of a major facilitator superfamily (MFS) transporter permease (LGG_RS00320) and 4.3-fold induction of the Hsp20-like molecular chaperone (LGG_RS03170). Conversely, a septation inhibitor protein (LGG_RS12050), *prsA* similar to LGG_RS10900, a molecular chaperone potentially involved in a late stage of protein export (Jakob et al., 2015; Jousselin et al., 2015), the NrdH-redoxin (LGG_RS07055), uracil transport (LGG_RS06995, LGG_RS07000, and LGG_RS07005) and peptide ABC transport system operon (LGG_RS07940, LGG_RS07945, LGG_RS07950, LGG_RS07955, and LGG_RS07960 were down regulated in AMC010 exposed to bile.

### *In vitro* growth and survival of *L. rhamnosus* exposed to simulated gastrointestinal conditions

**Figure 3:**
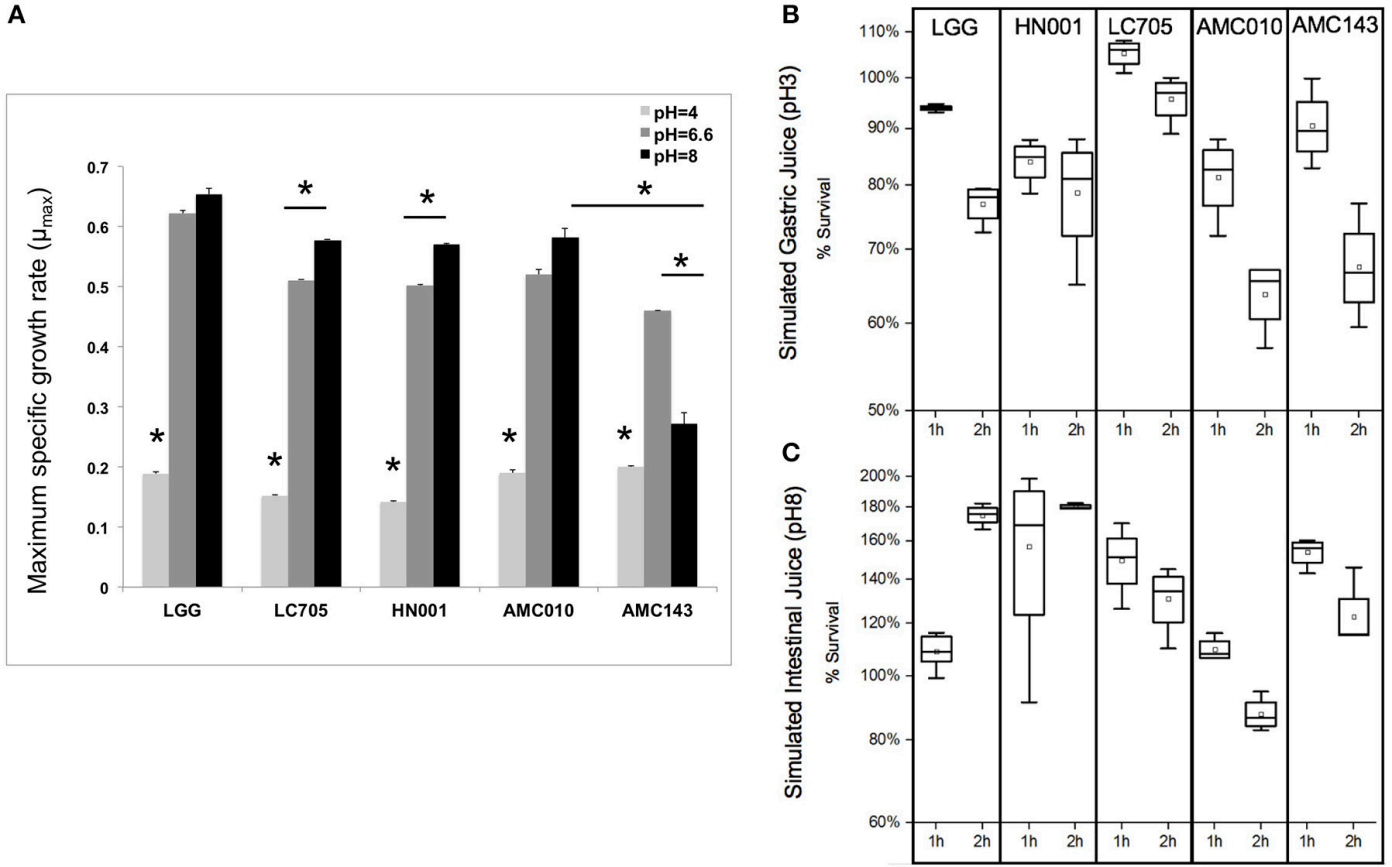
Growth and survival of *L. rhamnosus* under pH-stress conditions. **(A)** Growth rates were calculated for cells grown at pH 6.6 (MRS) pH4 (adjusted with HCl), or pH8 (adjusted with NaOH). (^*^p < 0.01). **(B,C)** Bacterial survival was assayed by plating and enumerating cells exposed to simulated gastric juice (NaCl, HCl, Pepsin pH3) or simulated intestinal juice (NaCl, HCl, Pancreatin pH8) for 1 or 2 h.

Under normal growth conditions (MRS broth, pH 6.6 at 37°C) LGG showed the highest growth rate among strains (0.63 h^−1^), while AMC143 had the lowest growth rate (0.48 h^−1^) with no significant differences between AMC010, Lc705, and HN001 (Figure 3A). While growth rates between strains were similar under normal conditions, there were distinct differences in growth phenotypes between strains when exposed to simulated gastrointestinal conditions. Growth rates of all strains were reduced approximately 5-fold at pH 4 (adjusted with HCl); however the strains isolated from dairy products, HN001 and Lc705, showed further reduced rates than the human-derived strains, AMC010, AMC143, and LGG (Figure 3A). As expected, survival of early-log cells exposed to simulated gastric juice (pepsin, NaCl, HCl pH3) was minimally impacted. No decrease in survival was observed for LGG, Lc705, or AMC143 after 1 h of exposure, while an approximately 15% decrease was observed for HN001 and AMC010 strains. After 2 h, Lc705 showed close to 100% survival, LGG and HN001 were reduced by approximately 20%, and AMC010 and AMC143 populations were reduced by 40% (Figure 3B).

Exposure of *L. rhamnosus* strains to MRS adjusted to pH 8 with NaOH showed nearly a 50% decline in growth rate only in AMC143. HN001 and Lc705 showed increased growth rates when grown in media at higher pH 8, while AMC010 and LGG showed no significant differences in growth rates compared to pH 6.6 (Figure 3A). When early-log cells were exposed to simulated intestinal juice (pancreatin, NaCl, NaOH pH8) (Charteris et al., 1998) cell numbers increased after 1 h to then decrease in all strains, except HN001 where survival was stable (Figure 3C).

**Figure 4:**
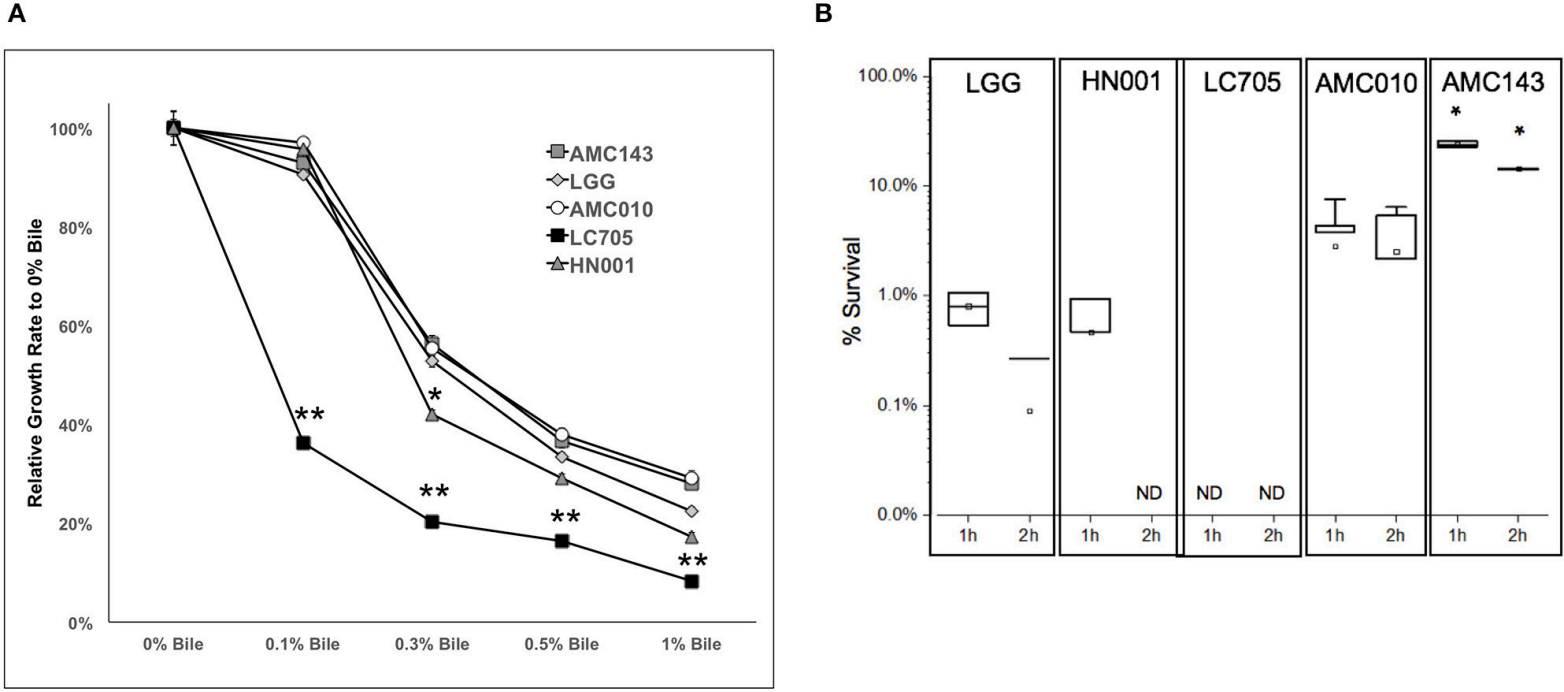
Growth and survival of *L. rhamnosus* under bile-stress conditions. **(A)** Growth rates relative to 0% bile were calculated for cells grown in MRS containing 0.1, 0.3 0.5, and 1.0% w/v oxgall. (^*^p > 0.05, ^**^p > 0.01). **(B)** Bacterial survival was determined by plating and enumerating colonies post exposure to 0.5% w/v oxgall in MRS devoid of any carbohydrate source for 2 h. ^*^Indicates significant difference between AMC143 and each other strain tested. (^*^p > 0.01, *ND* = No detected growth).

Finally, we sought to evaluate growth and survival of *L. rhamnosus* strains in the presence of bile. At sub-physiological concentrations of bile (0.1% w/v), the only strain that showed growth inhibition was Lc705 (60% growth rate reduction). As the concentration of bile in the growth medium increased to physiological levels (0.3% w/v), growth rates decreased by approximately 50% for AMC010, AMC143, and LGG, while HN001 and Lc705 were further inhibited (58 and 80% respectively) (Figure 4A). Lc705 showed significant decreased growth rates in all bile concentrations tested. As bile concentrations exceeded physiological levels (0.5% bile) growth rates decreased by over 60%. The growth inhibition observed at 1.0% w/v bile was greater for HN001 and Lc705 (83–92%) than for the strains of human origin (71–78%).

Exposure of *L. rhamnosus* to 0.5% bile resulted in a significant decrease in survival in all strains except AMC143. AMC143 exhibited nearly 20-fold higher survival rates compared to all other strains after 1 h of exposure to 0.5% bile. After 2 h, no HN001 colonies were detected, and the LGG and AMC010 populations were reduced by >95%. AMC143 however remained resistant to the antimicrobial effects of bile, with over 15% of cells surviving after 2 h of exposure (Figure 4B).

### The bile salt hydrolase (*bsh*) gene from *L. rhamnosus* AMC010 and AMC143

**Figure 5:**
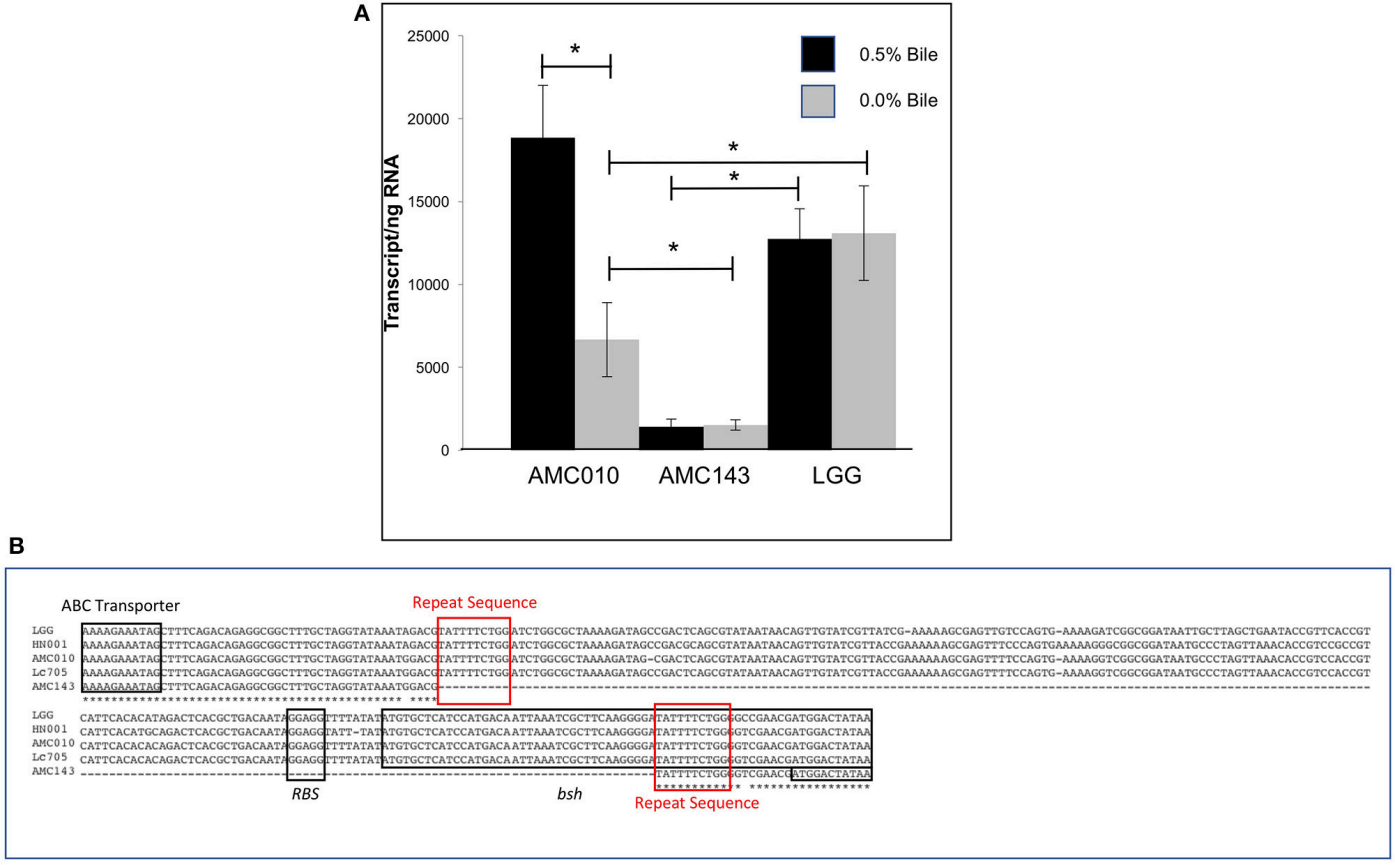
Bile salt hydrolase (*bsh*) expression and sequence analysis. **(A)** RTqPCR targeting *bsh* was performed on AMC010, AMC143, and LGG with and without acute bile treatment, (^*^p > 0.05). **(B)** Sequence alignment of *bsh* performed in CLUSTAL including annotations of coding regions and ribosome binding site.

Despite a highly conserved chromosomal architecture and gene sequence, the region upstream of the *bsh* gene in AMC143 contained a 208 bp deletion, eliminating a putative promoter region and ribosome binding site that is highly conserved in the other strains in the study (Figure 5B). Additionally, this deletion resulted in a truncation of the *bsh* gene. AMC143 showed significantly increased resistance to bile. To investigate if the deletion was responsible for the increased resistance of the strain to bile, we first assessed *bsh* expression levels in *L. rhamnosus* AMC143, AMC010, and LGG by RT-qPCR. Data showed variable expression of the gene in absence of bile. Upon bile exposure the *bsh* gene in AMC010 was induced approximately 4-fold, while expression of *bsh* homologs in AMC143 and LGG did not vary. Moreover, *bsh* expression was significantly lower in the presence or absence of bile in AMC143 (Figure 5A).

**Figure 6:**
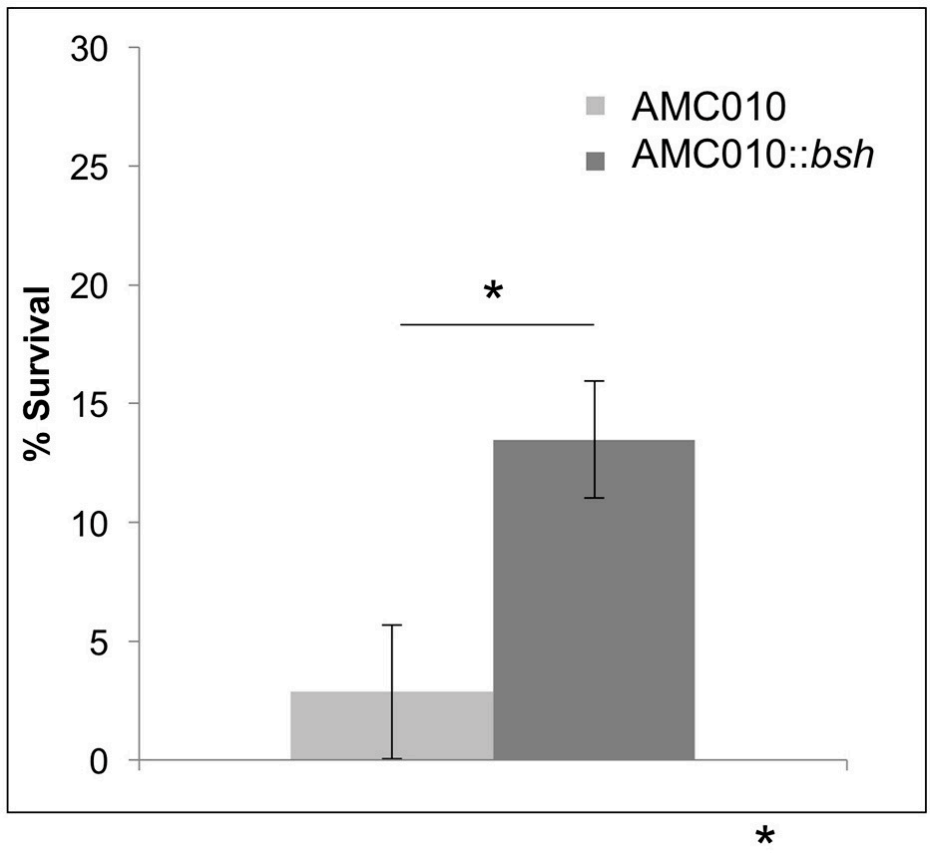
Survival of AMC010::*bsh* under bile-stress. Survival of AMC010::*bsh* was compared to wild type AMC010 by plating and enumerating colonies post exposure to 0.5% w/v oxgall (^*^p < 0.01).

To determine if inactivation of the *bsh* gene in AMC010 would replicate the bile resistant phenotype observed in AMC143, *bsh* was disrupted by insertion of the pFAJ-BSHi vector. Growth of the AMC010::*bsh* mutant under normal growth conditions was indistinguishable from the wild type strain. Survival and growth rates of the mutant were assayed under each stress condition previously performed in this study and compared to the wild type strain. When exposed to acid stress conditions, growth and survival of AMC010::*bsh* was identical to AMC010. Similarly, growth and survival at high pH was identical between the two strains (Supplementary Figure 1A). Growth rates of AMC010::*bsh* and AMC010 in varying concentrations of bile (0–1.0% w/v Oxgall) were similar in the presence of glucose (Supplementary Figure 1B); however, when exposed to high concentrations of bile (0.5% w/v Oxgall) without a carbohydrate source, survival of AMC010::*bsh* was enhanced 4–6-fold compared to the wild type strain (Figure 6), further suggesting a link between *bsh* expression and bile resistance in *L. rhamnosus*.

## Discussion

The future of personalized medicine will employ defined bacterial consortia to treat or prevent diseases according to host genotype, diet, and life stage, and the essential first step to develop such consortia is the extensive characterization of gastrointestinal organisms (Faith et al., 2010).

High-throughput *in silico* and *in vitro* analyses of gene-level variation across a large array of species in the human gut are useful as screening tools; however, detailed characterization of strains is essential to determine functional implications of strain variation in the complex ecosystem of the gut (Greenblum et al., 2015). Our study confirms that strain-level variability contribute significantly to microbial diversity within complex microbial communities (Zhang and Zhao, 2016). We compared genomic and physiological data of two novel strains of *L. rhamnosus* (AMC010 and AMC143) (Thompson et al., 2015; Arnold et al., 2017) to three characterized strains (LGG, Lc705, and HN001) to determine their response to simulated gastrointestinal stress.

One of the most striking physiological differences between the strains in our study was the growth inhibition of AMC143 exposed to alkaline conditions. Comparative genomic analysis revealed no major differences between genes previously identified as alkaline-stress response genes, with nucleotide identities ranging from 98.5 to 99.6% between strains. Despite this growth defect, the strain had comparable survival rates when exposed to simulated intestinal juice, suggesting that the growth defect is caused by arrest of cell division as opposed to cell death. Upon exposure to alkaline stress, expression levels of genes associated with growth inhibition (*divIC, ftsL*, and *minC*) were repressed in AMC010, while expression of the same genes in AMC143 was unaffected. Conversely, expression of the translation initiation factor *IF-3* was downregulated in AMC143 when exposed to alkaline conditions. We could speculate that this differential expression plays a role in the growth inhibition observed for AMC143. A previous study in *Lactobacillus plantarum* showed that expression of two endopeptidases (Accession numbers Q52071 and Q048X8) was reduced under alkaline conditions (Lee et al., 2011); however we failed to identify homologus genes significantly downregulated in AMC143. Further study of cellular response to alkaline pH is required to better understand the reasons for a growth defect in AMC143.

Analysis of genome sequencing data revealed a 208 bp deletion upstream of the *bsh* gene in AMC143 (Figure 1D). Sequence comparison between strains identified a repeat of 10 base pairs flanking the deleted region, suggesting that this deletion may have occurred in AMC143 through homologous recombination. Physiological assays showed that AMC143 was more resistant to bile-induced toxicity than the other strains tested, which led us to investigate the impact of *bsh* expression on bile toxicity. RTqPCR expression data showed a significantly lower *bsh* transcript count in AMC143 compared to LGG or AMC010, both in absence or presence of bile. A non significant induction of the *bsh* gene in AMC010 was also observed by mRNA sequencing data. Bile salt hydrolases have been associated to bile stress resistance in *Lactobacillus* and *Bifidobacterium* (Grill et al., 2000; McAuliffe et al., 2005; Lin et al., 2014) and implicated as a mechanism for host-microbe signaling (Zhou and Hylemon, 2014; Song et al., 2015; McMillin et al., 2016). These enzymes hydrolyze conjugated bile salts generating unconjugated bile acids, which often function as a signaling molecules to host cells and have strong anti-microbial effects (Sytnik et al., 1978; Grill et al., 2000; Schmidt et al., 2001; Kong et al., 2011), and an amino acid, which can be utilized by both host and microorganisms for protein synthesis (Ridlon et al., 2006, 2014; Patel et al., 2010). Studies have shown that BSHs do not always provide a survival advantage to bacteria exposed to bile (Fang et al., 2009), suggesting that BSH activity differs between microorganisms. In fact, lactobacilli encode variable *bsh* genes, each with unique substrates and activities (McAuliffe et al., 2005; Ren et al., 2011). To determine if *bsh* expression was correlated to lower survival upon bile exposure in our strains, we generated a mutant strain of AMC010 containing a disrupted *bsh* gene by site directed insertion (Lebeer et al., 2012). AMC010::*bsh* recapitulated the physiological phenotype observed in AMC143. These findings suggest that the unconjugated products of BSH activity were cytotoxic to the strains used in this study. Our survival assays were performed in MRS broth devoided of a carbohydrate source. In accordance to a previous report (Ziar et al., 2014), when survival experiments were performed in the presence of a carbohydrate source, bile cytotoxity was almost completely abolished for all strains (data not shown).

Strains of the same species derived from different environments are likely to have evolved different abilities to tolerate environmental stress (Douillard et al., 2013). Our data shows distinct physiological differences between strains isolated from human hosts (LGG, AMC010, and AMC143) and strains isolated from fermented dairy products (Lc705, HN001). When grown in alkaline conditions, dairy-derived strains exhibited accelerated growth, which correlates with studies done with other dairy-derived lactobacillus strains (Mojgani et al., 2015), while intestinal strains were either inhibited (AMC143) or unaffected (LGG, AMC010). Additionally, we found that while strains isolated from human hosts were able to grow with limited inhibition at sub-physiological levels of bile, the dairy isolate Lc705 was significantly inhibited even at very low bile concentrations (0.1%w/v). Moreover, survival of dairy-derived strains exposed to high bile concentrations was lower than intestinal strains, suggesting that the strain environmental origin may have driven evolution of their stress response pathways. Strains evolving in environments devoid of bile have no selective pressure to retain bile resistance genes, and these molecular mechanisms are lost over time. This phenomenon has been observed in dairy derived samples as well as in the oral microbiota (Douillard et al., 2013; de Barros et al., 2016).

Analysis of 16S rRNA sequencing data is unable to accurately resolve taxonomy at the species/sub-species level (Janda and Abbott, 2007) as single gene sequencing is insufficient to differentiate between similar species, especially in highly diverse and complex communities. Even with the best of what next generation sequencing has to offer, classical microbiology approaches are absolutely critical in understanding how microbial behavior and physiology correlate to genomic, proteomic, and transcriptomic data. As novel microbiota-derived interventions like new probiotics are being developed, it is important to keep in mind the magnitude to which seemingly minor genomic variability can result in changes to cellular physiology and thus probiotics efficacy.

## Author contributions

JA Designed and performed experiments, and wrote the manuscript; JS and JK Performed experiments; JR Performed bioinformatics analysis; MA Designed the experiments, advised JA, contributed to bioinformatic and statistical analyses of data and wrote the manuscript; All authors read and approved the final manuscript.

### Conflict of interest statement

The authors declare that the research was conducted in the absence of any commercial or financial relationships that could be construed as a potential conflict of interest.

## Supplementary material

Supplementary Figure 1 **(A)** Growth rates of AMC010::*bsh* in MRS at pH 4, 6.6, and 8. **(B)** Relative growth rate of AMC010::*bsh* in bile (0, 0.1, 0.3, 0.5, and 1.0% w/v oxgall).

Supplementary Table 1 Expression fold change of Lactobacillus rhamnosus genes under stress conditions.
