# Supplementary figures and images for: Intra-species Genomic and Physiological Variability Impact Stress Resistance in Strains of Probiotic Potential
Source: Front Microbiol. 2018 Feb 20;9:242. doi: 10.3389/fmicb.2018.00242 (PMC5826259; doi:10.3389/fmicb.2018.00242)

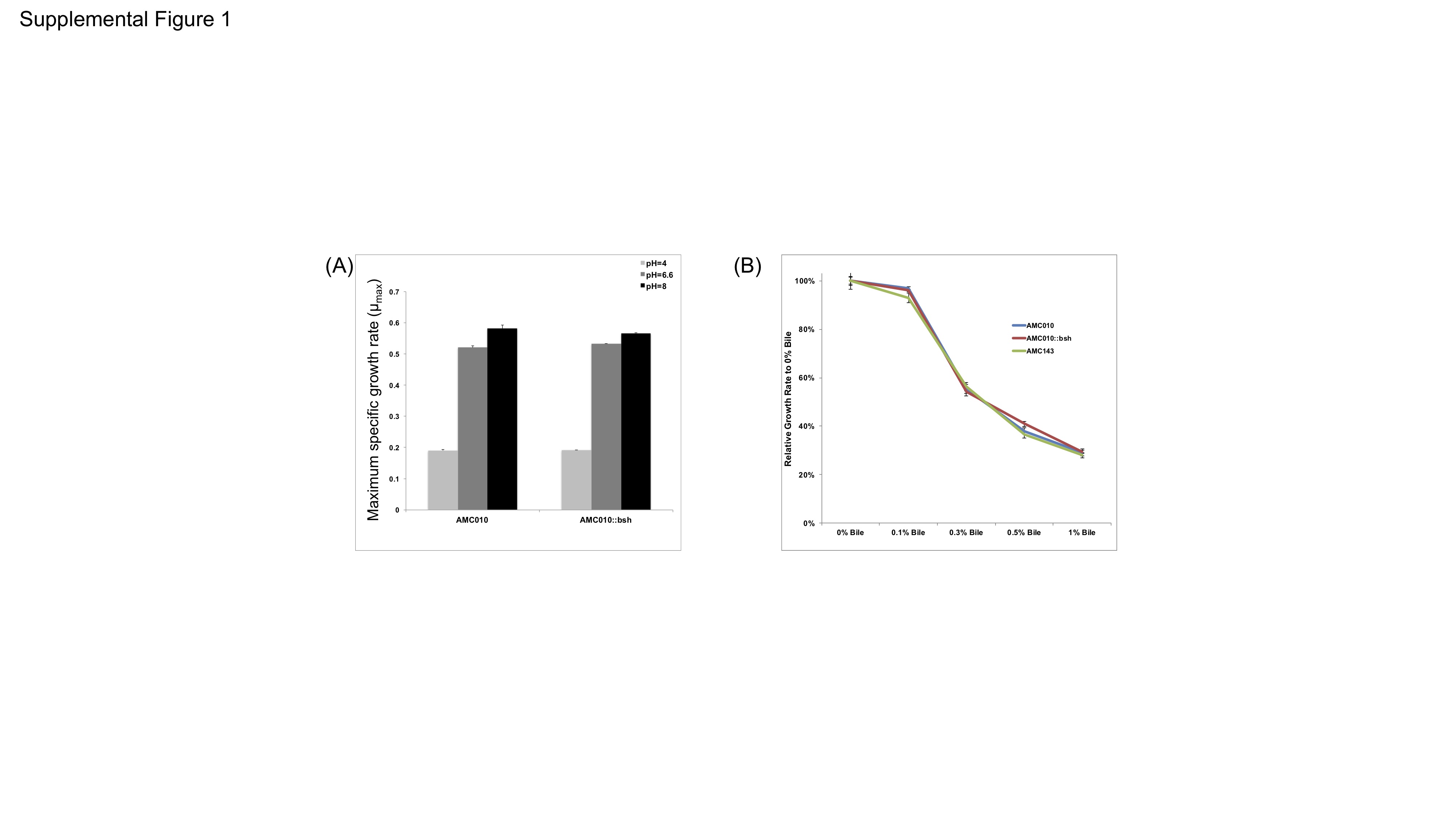

Supplement: Supplementary Figure 1 — (A) Growth rates of AMC010::bsh in MRS at pH 4, 6.6, and 8. (B) Relative growth rate of AMC010::bsh in bile (0, 0.1, 0.3, 0.5, and 1.0% w/v oxgall). [file Image1.jpg]
